# New Views of the Glomerulus: Advanced Microscopy for Advanced Diagnosis

**DOI:** 10.3389/fmed.2019.00037

**Published:** 2019-03-07

**Authors:** James M. Pullman

**Affiliations:** Division of Anatomic Pathology, Department of Pathology, Montefiore Medical Center and Albert Einstein College of Medicine, Bronx, NY, United States

**Keywords:** super resolution microscopy, electron microscopy, immunofluorescence, glomerular disease, podocyte, glomerular basement membrane

## Abstract

New technologies are ready to revolutionize glomerular imaging and significantly improve or replace immunofluorescence and electron microscopy, which have driven research and diagnosis of glomerular diseases for over 50 years. Advanced forms of transmission and scanning electron microscopy have revealed the detailed spatial relationships of the glomerular basement membrane, podocytes, and endothelial cells. These may be overshadowed by super resolution microscopy (SRM), which combines the advantages of immunofluorescence and electron microscopy, offers high resolution identification of specific molecules, and images large, physiologically relevant volumes of the glomerulus. Rapidity, ease of use and low cost with some types of SRM make them potentially suitable for routine diagnosis. SRM visualizes structures below the classical diffraction limit of conventional light microscopy by adding a time variable to either the illumination of the specimen, or to the fluorescence signal emitted by it. Ensemble techniques vary illumination and include Structured Illumination Microscopy (SIM) and Stimulation Emission Depletion Microscopy (STED). Single molecule localization techniques vary the light emission by fluorescence labels in the specimen, and include Photoactivated Localization Microscopy (PALM) and Stochastic Optical Reconstruction Microscopy (STORM). Technologies such as expansion microscopy and genetic labeling can also create effective super resolution imaging by non-optical, specialized preparation techniques. All technologies require dark field fluorescence and some require computer image analysis and reconstruction. Replicating successful application in other areas of biology, SIM, STED, and STORM have visualized normal and nephrotic disease podocytes, and have confirmed their appearances to be similar to those seen by electron microscopy, but with added new information on cell configuration and protein localization. STORM has also localized podocyte cytoskeleton and adhesion proteins, and glomerular basement membrane proteins at a resolution never before possible. These pioneering efforts show the promise of super resolution microscopy, and lay the groundwork for future study and new diagnostic tools for glomerular diseases.

## Introduction: The Current State of High Resolution Imaging of the Glomerulus

Advancement of our understanding and treatment of glomerular disease through improved methods of pathology diagnosis is a high priority, as recently recognized by the National Institute of Diabetes and Digestive and Kidney Diseases' Kidney Precision Medicine Initiative (1). High resolution imaging of the cells and macromolecular structures of the renal glomerulus plays a critical role in understanding the pathogenesis of its diseases as well as in their routine diagnosis. Currently researchers and diagnostic pathologists use bright field light microscopy (LM), immunofluorescence microscopy (IFM), and electron microscopy (EM) on tissue sections to probe the glomerular filtration barrier and its components the podocytes, filtration slit diaphragm, glomerular basement membrane (GBM), and endothelial cell, as well as other parts of the glomerulus and kidney. Of these techniques, only EM currently qualifies as high resolution, since it can identify subcellular and extracellular structural detail down to molecular levels, in the range of 1–10 nm. EM thus allows ultrastructural analysis of diagnostic abnormalities such as immune complexes, their substructure, and precise location; GBM thickening, thinning, splitting, and duplication; abnormal cellular morphologies and organelles, and the spatial relationships of these components (2).

To characterize fully abnormalities identified by EM in a way useful for diagnosis and treatment, it is also necessary to identify the proteins and other macromolecules involved. In diagnostic applications IFM is routinely used to identify and localize proteins of diagnostic importance at low resolution (~200 nm), such as the antibody and complement portions of immune complexes, but rarely antigens, cellular organelles, or the GBM. Variants of IFM, notably confocal microscopy, will improve resolution, but not to the level where important changes of cell morphology, such as podocyte foot process effacement, can be seen: EM is still necessary. Thus, IFM and EM are complementary and used together for routine diagnosis.

Despite the considerable successes achieved in understanding glomerular disease with IF and EM over the past 60+ years, there is a need for more versatile microscopies that can answer more questions, under a wider set of conditions, in less time, and with less expense than those currently in use. Several recently developed forms of EM and IFM may satisfy some of these needs, in particular super resolution microscopy (SRM), the collective term for the new technologies that transform IFM into a high resolution technique, with capabilities that may extend beyond those of EM. This review will first describe new forms of EM and their contribution to understanding glomerular structure, and then concentrate on SRM, describing different technologies and their contributions so far to imaging glomerular components, notably the podocyte and GBM where their need is greatest and where they have so far achieved success. The review will conclude with an assessment of the value and promise of the new microscopies for diagnosis of glomerular disease.

### Types of EM and Specimen Preparation

There are two major types of EM, both of which have been used in glomerular pathology: Transmission EM (TEM) and Scanning EM (SEM). Transmission EM (TEM), commonly used in diagnosis and research, focuses high energy (100 kV and higher) electrons with lenses to form images in a way similar to conventional light microscopy. It is most often used with thin sections of kidney tissue. Scanning EM (SEM), used almost exclusively in research, forms magnified images by scanning lower energy (10–50 kV) electrons focused into a narrow beam over the surface of tissues, and builds an image, point by point, analogous to confocal microscopy, from electrons that are scattered off a metal coating added to the tissue. This gives a three dimensional contour image in a volume and at a resolution between those of TEM and LM. SEM has been particularly useful in defining the complex structure of glomerular podocytes on the glomerular capillary basement membrane.

### Limitations of TEM and SEM

Although EM is the major high resolution imaging technology for biology, it lacks important features of LM and IFM. First, neither TEM nor SEM can easily identify specific proteins. ImmunoEM does combine the advantages of IFM and EM, but is cumbersome and limited by the requirement that antibodies bind specifically to antigens under the harsh specimen processing conditions necessary for EM (3). Second, the illuminating electron beam used with SEM and TEM damages and destroys biological macromolecules (4) thereby distorting or destroying image detail. Last, TEM can only image a very thin specimen. This is particularly important when attempting to visualize a three dimensional structure, such as a glomerular capillary curving through the glomerular tuft over a volume of ~10^3^ micrometers. A thin section used for conventional transmission EM (TEM) will easily include more than one capillary in a 10 × 10 micrometer cross section (the x-y plane), but will only include one five hundredth (100 nanometers) of the thickness (z axis) necessary to see the capillary in three dimensions. Useful, high resolution three dimensional information then comes only from fortuitous sampling such as a tangential view, or by three dimensional reconstruction from thin sections. The latter can be performed by stacking multiple images, or by tilting the specimen, and combining multiple images taken at different angles. Both methods are time consuming, difficult, and may require specialized equipment added to the instrument (5). Scanning EM (SEM) overcomes this problem by imaging surfaces over large areas and volumes, but at lower resolution than TEM and sacrificing morphologic or molecular details of cell interiors that TEM of sections provides (6). Because specimen preparation for SEM is also time consuming and equipment is not as available as that for TEM, SEM has not become a standard diagnostic tool. Therefore, there is a need for improved glomerular imaging with the resolution of TEM, the depth of field of SEM, and with all the advantages of IFM and LM.

### Contributions of Newer Forms of Scanning and Transmission EM (SEM and TEM)

It should be stressed, however, that EM continues to evolve and yield novel information about glomerular structure. Recent technologies have combined features of TEM and SEM that extend the resolving power of EM to specimen areas and volumes large enough to be of use in meaningful structure determination. Block face scanning microscopy (BF-SEM) has demonstrated the detailed and complex three dimensional structure of podocytes relative to each other and to the GBM (7). Of particular interest is the unique basal view of interdigitating podocytes, which shows the cross section of the filtration barrier immediately adjacent to the GBM and seen from that direction; almost all other 3 dimensional SEM images are views from the opposite direction, from the urinary space.

BF-SEM may be complemented by focused ion beam milling scanning microscopy (FIB-SEM) and Electron Tomography (goniometer tilt stage) TEM (5, 8). These techniques have provided large scale, high resolution information on the organization of glycocalyces of podocytes and endothelial cells. They have also demonstrated the intercellular relationships of podocytes to their nearest neighbors, to the subpodocyte space and to the GBM (7–9). However, these technologies remain highly specialized, and not readily available or adaptable to molecular localization techniques like IFM. The new light microscopy-based technologies of SRM hold more promise for uniting the resolving power of EM with the versatility and molecular identification abilities of IFM.

## Super Resolution Microscopy (SRM)

In the nineteenth century, Abbé and Rayleigh independently showed that the microscopes of the time could not image anything smaller than approximately half the wavelength of the illumination source. This condition is referred to as the diffraction limit, and for the shortest wavelength of visible light (violet, ~400 nm), it is 200 nm (10). This limit remains for contemporary light microscopes which use essentially the same optics (illumination and focusing lens technologies) of 150 years ago. However, in the latter twentieth and twenty-first centuries, several individuals devised optical theories and instruments based on them that image structures smaller than the diffraction limit (11). Advances in diverse areas of science and technology, including laser illumination, fluorescence labeling, and computing power facilitated what was formerly thought to be physically impossible, super resolution microscopy (SRM), and the 2014 Nobel Prize in Chemistry was awarded to 3 of the pioneers in this field (12). There are now several types of commercially available light microscopes that image structures and map macromolecules with the precision of electron microscopy, in their biologically relevant three dimensional environment (13). For the kidney, this means that SRM should be able to resolve normal and pathologic components of the glomerulus that are otherwise only visible by EM.

Super resolution may be achieved either with conventional, diffraction-limited light microscopes, such as confocal microscopes, under a small set of special specimen preparation conditions, summarized in the next section, or with microscopes designed to image below the diffraction barrier using a wider choice of specimen preparations, including those of conventional IFM (14). It is important to note that all forms of SRM are modifications of IFM, and cannot achieve super resolution with stains or preparations of conventional LM. This is because most LM is performed in “bright field” mode, which visualizes the specimen by the absorption and scattering of light by the stain molecules bound to it. This absorption of light provides only a small amount of contrast relative to the adjacent background, which is not much different from that of the specimen. The contrast provided by bright field LM is far less than that provided by IFM, which can detect single molecules by their fluorescence against a black background (15). Also, more light scatters from the specimen and background in bright field LM, which degrades resolution and impedes imaging overall, in particular the very complex techniques required by SRM. Fluorescence stains have the much higher contrast needed for molecular-level resolution, and emit light as self-luminous objects that may be focused without degradation from light scattering. Light scattering from tissue can still a problem with IFM, but may be minimized by specimen clearing, for thick tissues (16), and by optical filtering techniques, such as those as used in confocal microscopy (17). Note that super resolution techniques, like confocal microscopy, usually employ scanning methods rather than direct imaging with lenses, and may also benefit from specimen clearing (18).

### Diffraction-Limited SRM

Special specimen preparation techniques can enable resolution of structures smaller than the diffraction limit even when using diffraction-limited microscopes. There are three ways to accomplish this seemingly paradoxical feat. Expansion microscopy (EXM) physically rather than optically enlarges whole tissues by permeabilizing and then isotropically expanding them with a polymerizing gel so that structures smaller than the diffraction limit of light microscopy (200 nm) become larger than the diffraction limit and hence visible by IFM. EXM has been demonstrated to work in a wide variety of tissues, with both protein and RNA fluorescent labels (19). In the kidney, treatment of the whole organ with EXM techniques allows podocyte foot processes and filtration slits to be resolved by IFM [(18, 20); also see discussion in section Visualizing Normal Podocyte Foot Processes With SRM below].

The second technique, genetic labeling, is much more specialized and is restricted to animal models because it requires insertion of a reporter fluorescent molecule into a gene expressed only in the cell type of interest, e.g., podocytes, but not in all cells of this type. If one podocyte expresses the reporter molecule, but its neighbor does not there is sufficient contrast and resolution with confocal microscopy to resolve individual primary and secondary foot processes (21, 22).

There is also a computational method to extend resolution past the diffraction limit in otherwise diffraction-limited microscopes, super resolution radial fluctuations, which computationally analyzes temporal sequences of conventional microscope images, and generates a super resolution image (23).This intriguing technology has not yet seen application to the kidney, but if successful may turn out to be the easiest and least expensive way to perform useful super resolution imaging. It works by adding a time -dependent perturbation of the image to add the spatial information necessary for super resolution. This is also one of the principles of SRM discussed in the next section.

### Diffraction-Breaking SRM: Illumination- and Specimen-Based Systems

SRM usually refers not to the techniques just described in section Diffraction-Limited SRM, but to microscopy which breaks the diffraction barrier by adding time and space-dependent perturbations to the optics of the system. This may be accomplished by modulating either the microscope's illumination source or the light emitted from the specimen. Table 1 summarizes the SRM techniques discussed above and below.

**Table 1:** Comparison of commonly used SRM technologies.

**Imaging mode**	**Widefield**	**Confocal**	**EXM**	**SIM**	**STED**	**STORM**	**PALM**
Diffraction limited	Yes	Yes	Yes	No	No	No	No
X-Y resolution, nm	250	250	70	85–100	20–70	10–30	10–30
Z axis resolution, nm	500	475	200	300	40–150	10–75	10–75
3D imaging	No	Yes	Yes	Yes	Yes	Yes	Yes
Sample preparation time and effort	Less	Less	More	Less	More	More	More
Image processing needed	No	Yes	Yes	Yes	No	Yes	Yes
Image acquisition	Fast	Fast	Fast	Slower	Slow	Slow	Slow

There are two types of commonly used SRM that modulate the illumination source, structured illumination microscopy (SIM), and stimulated emission depletion microscopy (STED). SIM adds stripes with a periodicity close to the wavelength of illumination to the scanning beam. STED adds an annular pattern, but with two illumination sources. Both SIM and STED can be used with any type of fluorescent-labeled specimen. They image groups rather than single fluorescent molecules, and are therefore called “ensemble” techniques (24). There are also two types of commonly used SRM which modulate specimen light emission, stochastic optical reconstruction microscopy (STORM), and photoactivated localization (PALM) microscopy. Both require labeling the specimen prior to observation with fluorescing molecules that emit light for only very short time intervals, allowing an image to be built one point at a time. This category of SRM is referred to as “single molecular resolution techniques” or “pointillist” techniques (22). Both ensemble and single molecule resolution SRMs have been used to image the GFB. Each has advantages and disadvantages, and will be discussed in more detail.

#### Structured Illumination Microscopy (SIM)

SIM achieves super resolution by merging the spatial frequencies its striped illuminating beam with those of the specimen, mathematically described as convolution. All imaging can be described as convolution of microscope properties with the image, but SIM adds time and space varying illumination, which effectively magnifies the specimen as a Moiré pattern does. Structured illumination and collection of image data have to be repeated at several different angles to generate three dimensional information. From these multiple sets of data the original illumination pattern is removed by deconvolution, which yields a magnified image with double the spatial resolution. Another way to understand this improvement is to consider that the new image will have spatial frequencies that are the sum of the specimen and illumination frequencies (25).

A major advantage of SIM over any of the other super resolution technologies is that it does not depend on specialized molecular stains, and can use standard IF specimen preparation and staining techniques. This versatility is not only useful for experimental work, but also lends itself to routine diagnosis. It also outweighs the disadvantages of SIM, such as the only modest increase in resolution below the diffraction limit (a factor of two, to ~100 nm) as compared with other SRMs and the potential for artifacts generated by computed image reconstruction (26). SIM microscopes are also currently expensive, and require significant expertise to run (27). Nonetheless, initial results indicate sufficient resolution to allow SIM to substitute for EM in some types of glomerular disease diagnosis (see sections Visualizing Normal Podocyte Foot Processes With SRM and Changes in Podocyte Foot Processes in Nephrotic Disease as Seen by SRM below).

#### Stimulated Emission Depletion Microscopy (STED)

Like SIM, STED uses a modulated illumination source, in this case a double laser beam, in which the second laser superimposes an annulus of light on top of the first laser spot, and at a lower wavelength, in order to deplete fluorescence created by the first laser in the annulus. Since the first laser spot is at the diffraction limit of resolution, this effectively reduces the size of this spot, making it smaller, and effectively a probe well below the diffraction limit, ~50 nm, twice that of SIM. Disadvantages of STED include higher machine cost and complexity as compared with SIM and the need for higher intensity illumination, which requires photostable fluorescent stains and can damage and alter the specimen (28). The availability of STED instruments is also limited, and as a result STED has not seen as much application as SIM or STORM.

#### Stochastic Optical Reconstruction Microscopy and Photoactivateable Localization Microscopies (STORM and PALM)

STORM is the major single molecular resolution technique in common use (29). It and PALM use modulation of the fluorescent signals from the specimen to build an image one point at a time with detail well below the diffraction limit. Light emission from fluors in different parts of the specimen occurs randomly, over a short time period, with a very small probability of being simultaneous. Thus, two fluors separated by a distance less than the diffraction limit will not emit light together and therefore cannot interfere with each other. They will be detected separately during imaging and as each emission is detected its location can be directly mapped. With enough emissions separated in time as well as in space, an image can be built point by point. Since there is no diffraction effect between two points even if closer to each other than the diffraction limit, the resolvable distance between them can be arbitrarily small and limited only by fluor and target molecule size. For many proteins of interest, this is around 10 nm, and is the best resolution among all SRM. Two types of fluor, photo-switchable (on and off) or photoactivatable (on-only), will perform in this way. PALM uses either type of fluor, while STORM requires photo-switchable fluors. The resolution advantage over other super resolution technologies is a major advantage of PALM and STORM, but the need for specialized fluorescent specimen labels may inhibit their routine use.

## Application of SRM to Renal Pathology: the Podocyte

SRM has been applied, in proof-of-principle studies, to several areas of pathology, including rectal cancer (30), hemato-, and cytopathology (31), pemphigus vulgaris in dermatopathology (32), and breast cancer (33). Renal pathology is at least as good a candidate as these. It is uniquely suited for SRM since it already uses immunofluorescence microscopy and electron microscopy routinely, particularly for diagnosis of glomerular disease. Although all cell types in the glomerulus may be affected by disease, the podocyte is a good first choice to test SRM, since its evaluation always requires EM. EM shows podocyte foot process and other changes in patients with nephrotic diseases, and SRM should show these and possibly other new ones as well.

### History of Podocyte Microscopy: From Light to Electron Microscopy—and Back Again

The podocyte is also a good first choice for renal pathology by SRM, since it has a long history of characterization by LM and EM, and SRM is a fitting next step. Podocyte microscopy began with Malpighi in 1688, who used one of the earliest light microscopes, the “Galilean Lens,” to discover the glomerulus, which he related to the production of urine by the tubules of the kidney (34). Nearly 200 years later, in 1845, Gerlach described the epithelium covering the glomerular tuft, subsequently termed the visceral epithelium to distinguish from that lining Bowman's capsule (35). In 1915, using a silver impregnation technique analogous to those of Cajal with neural dendritic processes, Zimmerman observed a filamentous substructure protruding from the visceral epithelium cells wrapped around the glomerular capillary. He and others further confirmed and refined this observation between 1928 and 1933 [reviewed in Willis (36)]. The first independent verification of these remarkable light microscopic observations did not come until 1950 with the new technology of transmission electron microscopy (37). Although the thin processes first seen in the early twentieth century likely represent primary processes rather than the smaller pedicels and foot processes later seen by electron microscopy, it is amazing that the silver impregnation and hematoxylin-iron stains of the early twentieth century allowed their resolution at all. These observations might have laid the foundation for the histopathologic study of nephrotic disease, but instead were forgotten and the staining techniques at their basis replaced 30 years later by electron microscopy.

During the 1950's, multiple investigators studied the elaborate substructure of glomerular cells by electron microscopy, using a new processing and staining technique created for this technology, heavy metal staining of glutaraldehyde-fixed kidney tissue imbedded in plastic and thin sectioned (100 nm vs. 3 microns for light microscopy). They found that cytoplasmic extensions of the visceral epithelial cells interdigitated and formed part of the physiological glomerular filtration barrier, and were absent or greatly altered in nephrotic disease. These processes were named foot processes and the cells renamed podocytes (38–40). The next breakthrough in understanding podocyte substructure came in 1970 with the use of the scanning electron microscope (SEM) which gave a true three dimensional view of the podocyte, with a field of view nearly as large as light microscopy, but with resolution approaching that of transmission electron microscopy (5). SEM studies have shown more clearly than TEM the hierarchical branching structure of podocyte primary and secondary processes, their interdigitation between adjacent cells, and how they retract, or “efface” in nephrotic disease rather than “fusing” as has been previously concluded by some based on the limited thickness views afforded by TEM (41).

A return to the light microscope for the study of podocyte substructure came in 2012–13 with the use of genetic fluorescence labeling techniques in mice, which gave contrast sufficient to visualize individual podocyte foot processes by confocal microscopy (18, 20). Note that this is not a super resolution technology *per se*, but it improved the effective three dimensional resolution of light microscopy by eliminating out of focus parts of the image. The proof of success of this marriage of two technologies is that the observed podocyte substructure matches that seen by SEM and TEM. As discussed in section Diffraction-Limited SRM above, the new light microscopic technique of expansion microscopy has also successfully resolved podocyte foot process structure (17).

In summary, special staining, silver, and iron-hematoxylin in the early twentieth century and genetic labeling in the early twenty first century, has enabled some visualization of podocyte structure, even if not as detailed and useful as TEM or SEM have provided. TEM has persisted, until recently as the only useful and practical technique for visualizing podocyte structure in the routine diagnosis of glomerular disease. Now SRM is a candidate for this role.

### SRM Pathology of the Podocyte

#### Diagnosis of Nephrotic Disease With SRM

Initial SRM studies suggest two strategies for imaging podocytes in the glomerulus and diagnosing nephrotic disease. One is to stain proteins that are on or near podocyte surfaces to provide a one or two dimensional outline of their boundaries, and thereby show the disease-associated changes in podocyte foot processes seen in EM. A second strategy is to stain cytoskeletal proteins that fill the cytoplasm of the podocyte, generate a space-filling view of its primary and foot processes, and show how this changes in nephrotic disease. Staining glomerular basement membrane proteins simultaneously with either approach may also facilitate diagnosis by providing a spatial frame of reference. Table 2 summarizes the published superresolution studies of normal and diseased podocytes and glomerular basement membrane, as discussed in sections SRM Pathology of the Podocyte and Application of SRM to Renal Pathology: The GBM below.

**Table 2:** Summary of published SRM studies of the podocyte and glomerular basement membrane (GBM).

**SRM technology**	**Kidney tissue studied**	**Structure studied**	**Proteins studied**	**References**
STED	Rat, normal, and Heymann nephritis	Foot process and filtration slit	Nephrin, podocin, indirect IF	(18, 42)
SIM	Human biopsy, normal, and minimal change disease	Foot process	Podocin, indirect IF	(43)
SIM	Human, biopsy, normal, and minimal change disease	Foot process	Nephrin, indirect IF	(44)
STORM	Mouse, normal and Cdap-KO, Lamb2-KO, Adriamycin toxicity; Human biopsy, normal	Foot process, filtration slit, GBM	Nephrin, podocin, CD2AP, synaptopodin, α-actinin, actin, myosin IIA, integrin β1, agrin	(45)
STORM	Human, biopsy; mouse, normal, and mouse Alport model	GBM	Laminin, nidogen, collagen IV-α chains, agrin	(46)
EXM + confocal	Mouse, normal; human, nephrectomy	Foot process, filtration slit, GBM	Podocin, synaptopodin, podocalyxin, smooth muscle actin, collagen IV, agrin, tubulin	(20)
EXM + confocal	Human biopsy, normal, and minimal change disease	Foot Process, GBM	Actinin-4, synaptopodin, collagen IV, vimentin	(47)
EXM + STED	Rat and mouse, normal and Anti-GBM disease	Foot process, GBM	Podocin, nephrin, collagen IV	(18)

#### Visualizing Normal Podocyte Foot Processes With SRM

Using the first strategy above, four studies have so far characterized normal podocyte foot process structure. Three used indirect immunofluorescence with podocin, a protein that is present in a small area in the peripheral border of the podocyte foot process near the filtration slit diaphragm (42, 43, 45). One used nephrin, which is adjacent to podocin and an integral part of the filtration slit (44). Two of the studies used both nephrin and podocin staining (42, 45). All four demonstrated similar outlines of normal foot processes, with three different types of super resolution microscopy: SIM, STED, and STORM. Two studies validated their SRM results by correlation with TEM (43, 45).

**Figure 1:**
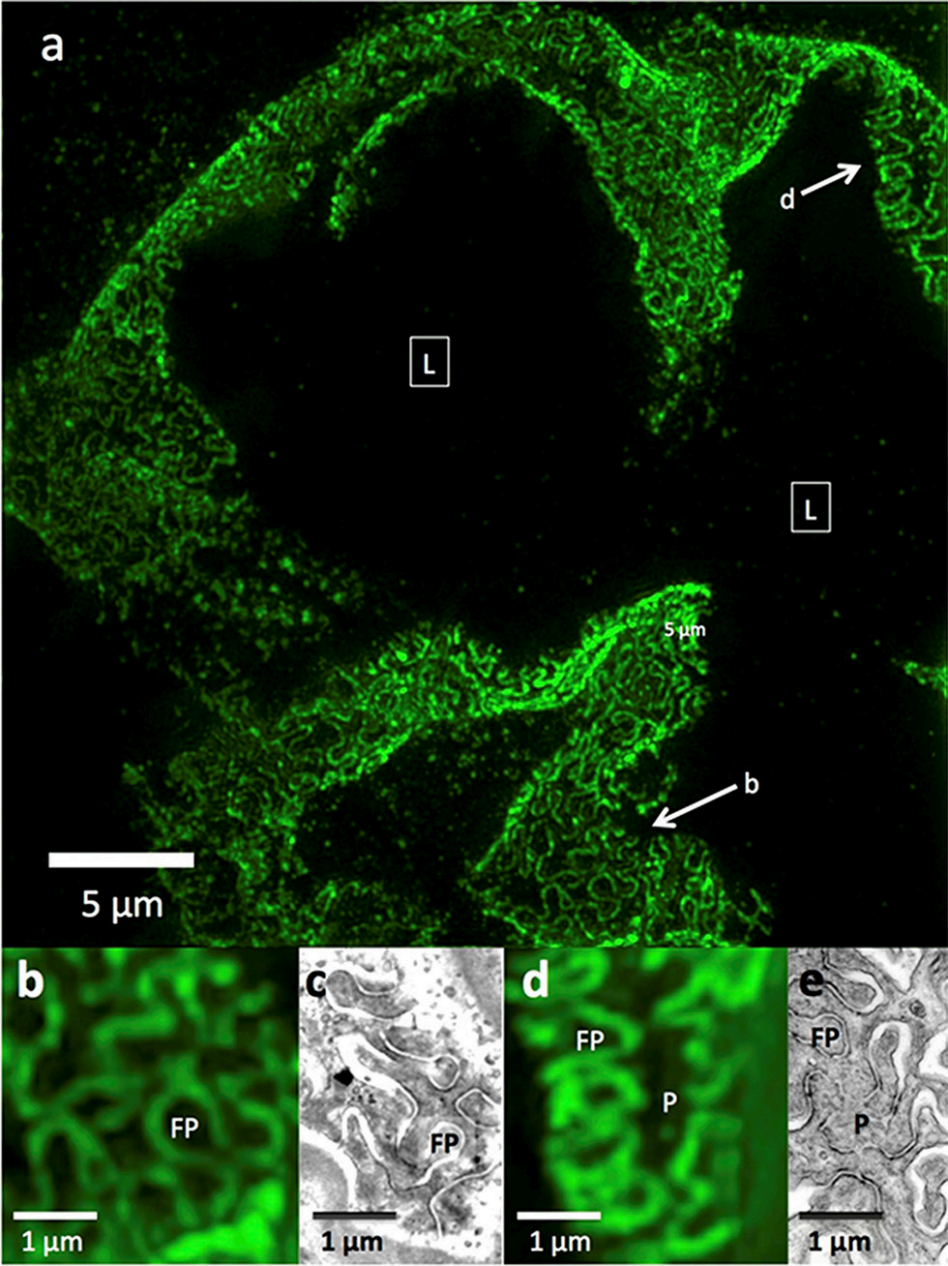
3D-SIM imaging of a FITC-podocin stained normal human glomerulus. Maximum intensity projection of a SIM z-stack (79 planes, 9.36 μm thick) at 100x. **(a)** Same region as shown in Figure 2. **(b)** Squares with (L) denote the capillary lumens. Image shows cross sectional and en face views of podocytes foot processes as they curve around capillary walls. Arrows point to well-resolved podocyte outlines. **(b,d)** Enlargements of two areas marked with arrows in **(a)**. FP, foot process; P, pedicel. **(c,e)** EM sections with similar en face views of podocytes at the same magnification as **(b,d)**. Reproduced with permission of the publisher from Pullman et al. (43).

In normal human and rat kidneys, foot processes were seen as a collection of sinusoidal lines ~200 nm width by 600 nm in length, often interlocking in tessellating patterns similar to those seen by SEM and TEM (Figure 1). These linear outlines were constrained to a curving, two dimensional surface which corresponded to the glomerular capillary wall. The foot process outlines correspond most closely to the “basal” view seen by Block Face Scanning Microscopy (6). They are also similar to the tangential view of podocytes occasionally found in thin section images of glomeruli, but are substantially larger (41). This size difference may be attributed to tissue shrinkage caused by fixation and processing for electron microscopy, in comparison with the hydrated state and milder tissue processing used in the preparation for SRM. Cross section views, obtained from single layers in the 3D SRM image, give an appearance of foot processes similar to that of most TEM images, and serve as further validation of SRM results (42). Using optical clearing as part of specimen preparation and STED as the SRM technology (42) or EXM alone (18, 47), the foot process outline becomes a double outline, with the filtration slit between the two outlines visible as an space 60–80 nm wide. This appearance corresponds to perfect interdigitation of foot processes. Nephrin, a slit diaphragm protein is in this space, as shown by double labeling (18, 42). However, in the published images, nephrin staining is not always between podocin layers but sometimes seems to override them. This may be a limitation of the resolution of STED or of EXM.

#### Changes in Podocyte Foot Processes in Nephrotic Disease as Seen by SRM

All four studies above compared normal to nephrotic disease foot processes using the outlines provided by staining with podocin, nephrin, or both. Nephrotic diseases studied included minimal change disease in human biopsies, Heymann nephritis in a rat model, Lamb-2, and Cd2ap mouse knockout models and an Adriamycin injury mouse model. In all instances the podocyte outlines showed enlargement, with a variable increase in the average foot process width, correlating with the change in podocyte cross section width seen by TEM.

A difference seen with STED but not SIM is that the podocin double contour of normal foot processes is lost in multiple segments in nephrotic disease, specifically in the Heymann nephritis rat model. Lines marking the borders of adjacent foot processes appear to be fused over short stretches that alternate with intact double contours (43). Whether this is a true fusion of adjacent podocytes or is caused by the loss of resolution has yet to be determined.

**Figure 2:**
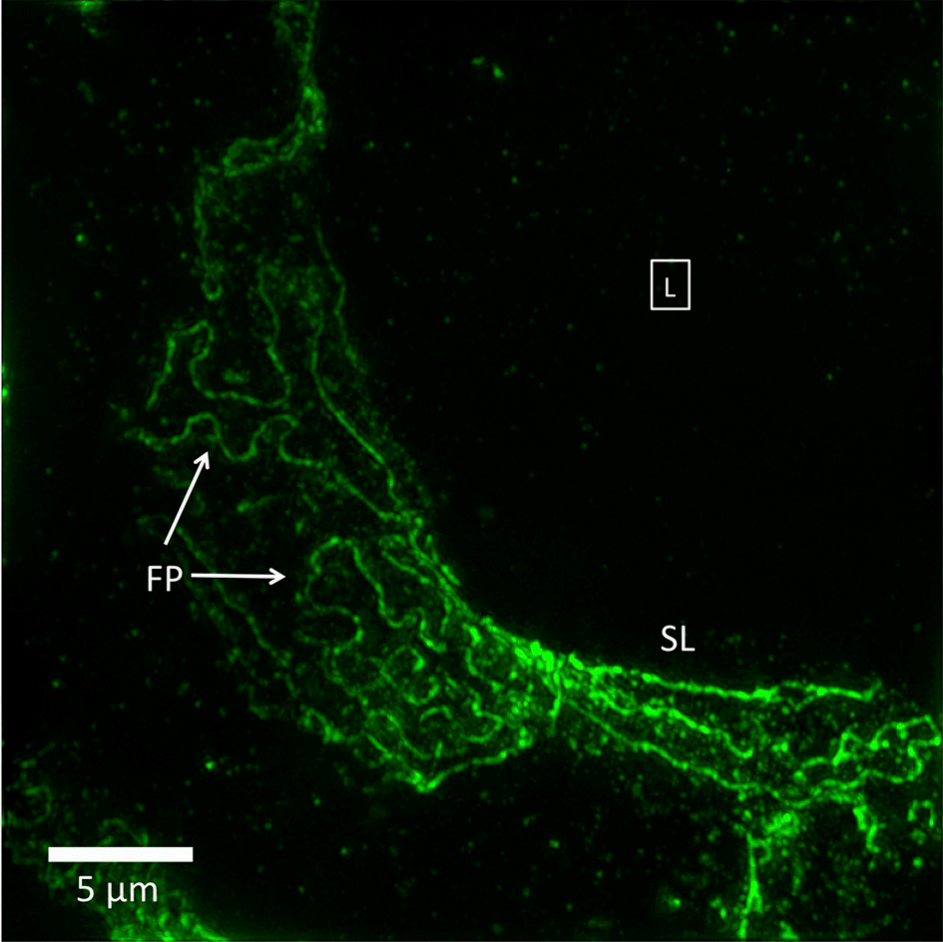
3D-SIM image of a FITC-podocin stained glomerular capillary from a patient with nephrotic syndrome (minimal change disease). Maximum intensity projection of a SIM z-stack (100x; 67 planes, 7.92 μm thick). Foot processes (FP) appear variably enlarged and sparser than the normal ones in Figure 1. Straight or minimally curved lines (SL) represent completely effaced foot processes. Square with (L) denotes the capillary lumen. Reproduced with permission of the publisher from Pullman et al. (43).

Not specifically noted by any study is that the nephrotic disease pedicel-foot process unit is sometimes increased in size but maintains the same linear proportions of pedicel to foot process, as though it has been magnified. Another type of change, possibly representing more severe nephrotic disease, is a complete or near-complete loss of foot process curvature, corresponding to the total effacement seen by TEM. In SIM images, this results in long runs of almost parallel lines, which make sharp turns in a zigzag fashion. The borders of podocytes from adjacent cells are separated, never interdigitate or touch, and bear no resemblance to foot processes (Figure 2). These have never been seen by SEM or TEM, and their significance is yet to be determined.

In the one SIM study using nephrin as a marker of podocyte outlines, the nephrin content of nephrotic human kidneys did not appear to be diminished in comparison with normal kidneys, as measured by staining intensity, or in location, and distribution. The nephrin outlines of podocyte foot processes were instead similar if not identical to those of podocin seen in the other studies (40). This is somewhat surprising, given that the nephrin-containing structure, the filtration slit, disappears from EM images in nephrotic disease. This paradox may be explained by a study using STORM which showed that in cross section, nephrin was present close to the GBM in normal controls and moved away from the GBM in three animal models of nephrotic disease (41). This suggests that although filtration slits containing nephrin are lost in nephrotic disease, the component proteins translocate only slightly, to the inside of the foot process cytoplasm close to their former extra-cytoplasmic location. The difference between the SIM and STORM results may be due to the superior resolution of STORM, or less likely, to the use of animal models vs. human biopsies.

#### The Cytoskeleton in Normal and Nephrotic Podocytes

There is one published super resolution microscopy study on the cytoskeleton of the podocyte (41). The authors used STORM to show the rearrangement in nephrotic disease of cytoskeletal proteins myosin IIA, actin and synaptopodin between two cytoplasmic compartments: the podocyte cell body-major processes, and the pedicels-foot processes. In normal podocytes, synaptopodin localizes to the pedicels and foot processes, adjacent to the GBM, and actin and myosin IIA to the primary processes and cell body, away from the GBM. In nephrotic disease, synaptopodin moves from the secondary and foot processes into the cell body, as the foot processes retract during the process of effacement. Actin and myosin IIA move toward the base of the podocyte foot processes, where they rearrange to form actin-myosin sarcomere-like structures. These are seen by TEM as dense filamentous mats in cytoplasm of effaced podocytes, close to the GBM. The authors demonstrated this with 3 mouse models mentioned above (Cd2ap-KO, Lamb2-KO, and Adriamycin) and 3 examples of human nephrotic disease (minimal change disease, FSGS, and diabetic nephropathy). The STORM images in two dimensional cross section show these changes most clearly; three dimensional images were taken with an Airyscan confocal microscope, which although not a true SRM, is able to form images at a resolution intermediate between confocal and SRM. The differences between the normal and nephrotic cytoskeletal protein distribution was present but difficult to see in the three dimensional view, either because of the decreased resolution of Airyscan, or because of inherent difficulties in changes in space-filling proteins.

## Application of SRM to Renal Pathology: the GBM

There is only one SRM study of the GBM, using STORM with human and mouse models to localize GBM proteins in cross section, including adjacent proteins in the endothelium and podocyte (46). This was more difficult to accomplish than SRM of podocytes because the higher density of the GBM caused unwanted light scattering and background fluorescence with the loss of high resolution. A specimen preparation technique created for another purpose, the Tokuyosu method, solved this problem and also enabled validation of the STORM results by EM and immunoEM. Immuno-localization by STORM showed the arrangement of major constituents of the GBM. Laminin was distributed across the GBM in 2 layers in the mouse and 3 layers in the human GBM, nidogen at the center, collagen α3α4α5 IV next and agrin on the sub-endothelial and sub-epithelial peripheries. Mouse and human GBMs had similar arrangements of these proteins, but the human GBM had a more complex substructure, not surprising considering it is three times the thickness of the mouse GBM. Notably lacking was an examination of differences between normal GBM protein distribution and that in nephrotic disease. The authors did look at a single glomerular disease involving GBM structural changes, a mouse model of Alport syndrome. In this they demonstrated a change in collagen α1α1α2 (IV) distribution from two layers to a single, more centralized layer.

## Summary and Discussion

This review discusses the first studies of glomerular histology and pathology obtained with new EM and SRM technologies, whose contribution to biomedical imaging is the ability to perform high resolution imaging and protein mapping with immunofluorescence over a very large volume. This is exactly what is needed to advance the study and diagnosis of glomerular disease. The most significant results so far are for the podocyte, with 5 papers on SRM of normal and nephrotic disease published in the past 3 years (18, 41–43, 45). The results of these studies are remarkably consistent and in agreement with each other, except for some minor, but interesting discrepancies discussed below. Some of the SRM findings also correlate with those of newer SEM technologies, notably the podocyte basal view (8). This view can be seen in podocyte images, all remarkably similar, made with different types of SRM, including SIM, STED, STORM as well as BF-SEM and conventional TEM. The distribution of podocyte-associated proteins podocin and nephrin is similar as viewed with SIM, STED, or STORM and appears as predicted by immunoEM localization (46, 48).

The basal podocyte views in the SIM and STED views show an interesting difference: the podocin along the cell border is resolved as two separate lines by STED but not by SIM. Since two foot processes from adjacent podocytes are closely juxtaposed along these borders, the STED view is more accurate, and is validated by the localization of nephrin in the space between the two borders, where it is predicted to be. This difference between the STED and SIM images of podocin labeled foot processes may be due to the superior resolution of STED. That this is a resolution issue is supported by the observation that expansion microscopy followed by confocal IFM (a non-SRM) also resolves two podocyte borders (18).

The alterations in podocyte foot process morphology in nephrotic disease are also consistent among SIM, STED, STORM, and TEM. The podocyte cell borders, with one important exception discussed later, show foot processes and pedicels (or secondary processes) that are larger but approximately the same shape as normal. The loss in curvature in the enlarged podocytes is likely a manifestation of effacement, or foot process retraction. One interesting change in nephrotic podocytes, seen only in a STED publication, is that the double border marking adjacent foot processes and surrounding the slit diaphragm (where nephrin is present) partially disappears (18). Whether this is an imaging artifact or represents true foot process fusion, rather than effacement remains to be determined. The other nephrotic disease change in foot process outlines was a total loss of normal foot process and pedicel morphology and its replacement by long, zigzagging lines, which could represent a more severe response of the podocyte to nephrotic injury, but is currently of unknown significance. These “strung out” and folded foot process borders are so large that they can only be seen in a 3D SIM view that spans a significant part of a glomerular capillary (41). Because of changes like this, which need to be more fully explored, it may be premature to adopt simple linear measurements of foot process diameter as a measure of effacement or disease severity, as has been proposed (45).

One study also used STORM to study the change in nephrotic syndrome of protein distribution in the podocyte surface, cytoskeleton and slit diaphragm (42). It showed, in cross section, that nephrin clearly moved away from its normal location adjacent to the GBM to a more spread out location within the podocyte cytoplasm, and confirmed an earlier observation made by TEM (49). A SIM study did not show a change in nephrin distribution in nephrotic disease, either because the resolution of SIM was not sufficient (while STORM is), or because the en face view in the SIM study would not show a redistribution of nephrin that was at right angles to the image plane. The changes in podocyte cytoskeletal proteins in nephrotic syndrome seen by STORM were easy to see in two dimensional cross sections, but the three dimensional images made with a lower resolution microscope, the AiryScan were difficult to interpret. This may only reflect an inherent difficult in visualizing protein distributions that fill three dimensional space in the cytoplasm (actin, myosin IIA, synaptopodin) compared with proteins confined to a line on the cell surface (podocin, nephrin).

The only SRM study of a glomerular component other than the podocyte, the GBM, was a technical tour do force that mapped the major proteins within it. However, it only compared normal with one mouse model of Alport's nephritis, and omitted nephrotic disease (44). The ability to detect changes in the composition or arrangement of the GBM in glomerular disease would be of great value and deserves further consideration.

## Future Directions for SRM in Glomerular Disease Diagnosis

Diagnosing nephrotic disease by examining podocyte alterations is only one aspect of electron microscopy of the glomerulus. EM is routinely used to evaluate abnormalities in endothelial and mesangial cells, cytoplasmic organelles, and to localize immune complex deposits to specific locations in the glomerulus, as well as to detect substructure within them. Should we expect SRM to replace TEM, or merely to supplement it? Or can it provide new types of diagnosis that TEM is not capable of? Exploratory work with SRM needs to be done in all these areas, including podocyte and GBM pathology.

Regarding instrumentation, the studies reviewed here show that not all SRMs are equal. STED and STORM are higher resolution and show more detail in podocytes than SIM, while EXM may eliminate the need for SRMs altogether, as might super resolution radial fluctuations, since both work with diffraction limited microscopes, which are more abundant, less costly and easier to operate than SRMs.

Clearly much more exploration and validation of SRM findings in the glomerulus is needed to render it a useful diagnostic tool. The groundwork has been set, and the choices are many.

## Author Contributions

The author confirms being the sole contributor of this work and has approved it for publication.

### Conflict of Interest Statement

The author declares that the research was conducted in the absence of any commercial or financial relationships that could be construed as a potential conflict of interest.
